# Complete Genome Sequence of vB_EcoP_SU7, a *Podoviridae* Coliphage with the Rare C3 Morphotype

**DOI:** 10.3390/microorganisms9081576

**Published:** 2021-07-24

**Authors:** Shazeeda Koonjan, Callum J. Cooper, Anders S. Nilsson

**Affiliations:** 1 Department of Molecular Biosciences, The Wenner-Gren Institute, Stockholm University, 10691 Stockholm, Sweden; anders.s.nilsson@su.se; 2 School of Pharmacy, Pharmaceutical and Cosmetic Sciences, Faculty of Health Sciences and Wellbeing, University of Sunderland, Sunderland SR13SD, UK; Callum.Cooper@sunderland.ac.uk

**Keywords:** *Kuravirus*, C3 morphotype, *Podoviridae*, phage, genome annotation

## Abstract

Enterotoxigenic *Escherichia coli* (ETEC) strains are an important cause of bacterial diarrheal illness in humans and animals. Infections arising from ETEC could potentially be treated through the use of bacteriophage (phage) therapy, as phages encode for enzymes capable of bacterial cell lysis. vB_EcoP_SU7 was isolated from the Käppala wastewater treatment plant in Stockholm, Sweden, and propagated on an ETEC strain exhibiting the O:139 serovar. Transmission electron microscopy confirmed that vB_EcoP_SU7 belongs to the *Podoviridae* family and has the rare C3 morphotype of an elongated head. Bioinformatic analyses showed that the genome was 76,626 base pairs long and contained 35 genes with predicted functions. A total of 81 open reading frames encoding proteins with hypothetical function and two encoding proteins of no significant similarity were also found. A putative tRNA gene, which may aid in vB_EcoP_SU7’s translation, was also identified. Phylogenetic analyses showed that compared to other *Podoviridae*, vB_EcoP_SU7 is a rare *Kuravirus* and is closely related to *E. coli* phages with the uncommon C3 morphotype, such as ECBP2, EK010, vB_EcoP_EcoN5, and vB_EcoP_SU10. Phage vB_EcoP_SU7 has a narrow host range, infecting 11 out of the 137 *E. coli* strains tested, a latency period of 30 min, a burst size of 12 PFU/cell, and an adsorption rate of 8.78 × 10^−9^ mL/min five minutes post infection. With a limited host range and poor infection kinetics, it is unlikely that SU7 can be a standalone phage used for therapeutic purposes; rather, it must be used in combination with other phages for broad-spectrum therapeutic success.

## 1. Introduction

*Escherichia coli* is a common Gram-negative bacterium that is often found in the gastrointestinal tract of mammals and humans [1]. While there are a vast number of strains of *E. coli* that live as a normal part of the commensal microflora (estimated to be around 0.1% of the commensal microflora in humans) [2,3], there are a number of strains that are capable of causing disease [4]. Enterotoxigenic *E. coli* (ETEC) strains are among the major causative agents behind diarrheal diseases in low- and middle-income countries (often referred to as traveler’s diarrhea) and in animal farming [5,6]. ETEC is characterized by its ability to produce heat-labile and/or heat-stable enterotoxins, which result in the intestinal lining secreting excess fluid and causing diarrhea [7].

ETEC infections are commonly treated with different antibiotics, such as fluoroquinolones (most used) and ciprofloxacin [6,8]. However, due to the ongoing widespread misuse of antibacterial agents, bacteria such as ETEC have developed resistance against commonly used antibiotics [8,9]. As a result, there has been renewed interest in using bacteria-specific viruses, otherwise known as phages, as an alternative to treat antibiotic-resistant infections since they encode for proteins associated with bacterial cell lysis. Infections caused by a single species, such as *E. coli*, as well as those caused by multiple bacteria have the potential to be treated with a cocktail comprised of different phage combinations [10], the efficacy of which is dependent on individual phage dosing, size, and virulence [11]. For phage therapy to be effective, new phages have to be identified and characterized. Understanding the genome composition and phylogeny of a phage can shed light on its ability to infect and adsorb using different bacterial receptors, such as the O-antigen in *E. coli* species, as well as on their infection pharmacology and the development of phage-bacteria resistance. In this study, we present the isolation and genome annotation of vB_EcoP_SU7 (SU7), a *Podoviridae* coliphage with the C3 morphotype.

## 2. Materials and Methods

### 2.1. Bacterial Strains and Growth Conditions

The bacterial strains used for host range analyses were provided as follows: the *E. coli* ECOR standard reference collection [12] was kindly provided by Diarmaid Hughes and Dan Andersson (Uppsala University, Sweden) and the ETEC strains were provided by Martin Weiss Nielsen (Danmarks Tekniske Universitet Veterinærinstituttet, Kongens Lyngby, Denmark) and Åsa Sjöling (Karolinska Institute, Solna, Sweden). SU7 was propagated on the bacterial strain it was originally isolated on (ETEC exhibiting O:139 serovar; ETEC7). Bacterial cultures were grown in Miller’s lysogeny broth (LB; Neogen, Lansing MI, USA) with shaking at 150 RPM or on tryptone yeast agar (TYA; Biolife Italiana, Milano, Italy) at 37 °C.

### 2.2. Phage Propagation and Purification

SU7 was previously isolated from the Käppala wastewater treatment plant located 15 km East of Stockholm, Sweden, in November 2016. For phage enrichments, fresh LB media was inoculated with 100 µL of overnight bacterial cultures and allowed to grow to the mid-log phase at 37 °C with shaking or until the optical density at 600 nm (OD_600_) was 0.6. SU7 was enumerated using the agar overlay (OA) method with 65% *w*/*v* (22.75 g/L) TYA as previously described [13,14]. Concentrated stocks of SU7 were produced using a modified polyethylene glycol (PEG) precipitation protocol [15]. In brief, crude phage lysate suspensions were centrifuged at 3864× *g* for 10 min and the supernatant passed through a sterile 0.45 µm syringe filter (Sarstedt Filtropur, Nümbrecht, Germany). Phages were precipitated by adding solid NaCl and PEG8000 (Acros Organics, Fisher Scientific, Schwerte, Germany) to the partially purified suspension to final concentrations of 1 M and 10% *w*/*v*, respectively, and then stored at 4 °C for two weeks. Following refrigeration, phages were centrifuged at 11,000× *g* for one hour at 4 °C, the pellet re-suspended in 50 mL phosphate-buffered saline (PBS), pH 7.4, and the phage content determined using the OA method.

### 2.3. Plaque Morphology Determination

SU7 plaque morphology was determined using ETEC7 as the host bacterium and the OA method [14]. In short, SU7 phage stock was serially diluted 1:10 in PBS. Three mL of OA was inoculated with 100 µL of overnight host bacterium culture (approximately 10^8^ CFU/mL) and 100 µL of 10^9^ serially diluted SU7. The OA was poured over the surface of a pre-prepared TYA plate and incubated for approximately 18 h at 37 °C. Plaques were imaged using a Samsung SM-G950U camera. Twenty diameters were calculated using ImageJ software [16].

### 2.4. Host Range Analysis

The host range of SU7 was determined using the spot test assay as previously described [17]. In brief, inoculated OA was prepared by adding 100 µL of overnight bacterial culture to 3 mL of 65% TYA, which was gently swirled and poured over the surface of a pre-prepared TYA plate. A working solution of the SU7 phage was diluted in PBS to a final concentration of 10^5^–10^6^ plaque-forming units (PFU)/mL and the phage content was determined by the OA method. Prepared bacterial plates were then inoculated with approximately 10 µL of the diluted SU7 phage suspension using a stamper. The spots were allowed to dry at room temperature, plates incubated at 37 °C overnight, and then assessed for its ability to produce a plaque. Host-range experiments were replicated in triplicate unless otherwise stated in the text.

### 2.5. Transmission Electron Microscopy (TEM)

PEG purified SU7 (1.1 × 10^11^ PFU/mL) was negatively stained with 1% *w*/*v* uranyl acetate [18] and visualized on a TECNAI G2 Spirit Bio TWIN, 80 kV (FEI Company). Dimensions of eight SU7 virions were measured at 13,000× magnification and analyzed with ImageJ [16].

### 2.6. SU7 Infection Kinetics

The latency period, burst size, and adsorption rate constant for SU7 were determined using the modified one-step growth curve protocol used by Koonjan et al. (2020) [19]. In brief, 50 mL of LB was inoculated with 50 µL of ETEC7 and incubated at 37 °C with shaking until the bacteria reached the mid-log phase (OD_600_ 0.6). Once OD_600_ 0.6 was reached, the bacterial suspension was removed; the final volume of the bacterial suspension used for the experiment was 44.982 mL. A total of 18 µL of the SU7 phage stock (1.50 × 10^10^ CFU/mL) was added to the mid-log-phase bacteria (approximately 5.03 × 10^7^ CFU/mL) at a multiplicity of infection (MOI) of 0.12 and mixed by swirling (T = 0). Aliquots of 1 mL, withdrawn every five minutes for an experimental duration of 65 min, were centrifuged at 6000× *g* for one minute. Using the supernatant, 1:10 serial dilutions in PBS were done to determine the phage content during each time point. All experiments were performed in triplicates. The adsorption rate constant was determined using the following formula shown below, where *N* is the bacterial density, *P_o_* and *P* are the starting and ending phage titers, *k* is the adsorption rate constant, and *t* is the time in minutes over which adsorption occurs:

k=−ln(P/Po)/Nt

It should be noted that the adsorption rate constant was determined using only two time points (T = 0 and T = 5). The burst size was calculated by dividing the phage titer after the first burst (approximately at 35 min) with the number of adsorbed phages (initial phage concentration at T = 0 minus phage concentration at T = 5).

### 2.7. Phage DNA Extraction

SU7 DNA was extracted from suspensions containing a minimum of 1 × 10^8^ PFU/mL using the Norgen Biotek phage DNA isolation kit (Nordic BioSite AB, Täby, Sweden) according to the manufacturing instructions but with an additional DNAse I treatment. Before carrying out the sequencing, the phage DNA concentration was quantified by fluorometry on a Qubit 2.0 (Invitrogen, Thermo Fisher, Stockholm, Sweden) and the purity assessed by gel electrophoresis.

### 2.8. Genome Sequencing and Bioinformatics of the SU7 Genome

SU7 genome library preparation and sequencing on the Illumina Miseq platform, with paired-end 300 base-pair (bp) reads using a V3 600-cycle kit, were carried out by Eurofins (Ebersberg, Germany). The 718,152 reads in the phage index were quality controlled using FastQC version 0.11.8 [20]. Reads were assembled and evaluated using SPAdes in careful mode [21] and QUAST version 4.5.4 [22], respectively. Open reading frames (ORFs) and genes were predicted using Prokka version 1.14.5 on the Galaxy@Pasteur platform and Glimmer3 prediction in the Geneious 6.1.8 software package [23,24,25,26,27,28]. Inferred amino acid sequences were compared against the National Center of Biotechnology Information (NCBI) non-redundant protein sequences database restricted to *Caudovirales* using the Basic Local Alignment Search Tool (BLAST) BLASTx software [29]. Hypothetical bacterial σ^70^ and phage promoter regions were found using BPROM [30,31] and PhagePromoter on the Galaxy@GalaxyDockerBuild platform [25,32,33], respectively. Rho-independent terminators were found using the ARNold web server [34,35]. Ribosomal binding sites (RBS) were identified based on the Shine–Dalgarno sequence AGGAGG (mismatches allowed included AAAA, AAGG, AGAGAGA, AGGA, AGGAGA, AGGG, GAGA, GAGG, GAGGA, GCGG, GGA, GGAA, GGAG, GGAGA, GGAGG, and GGGAA) in the untranslated regions approximately 5–15 nucleotides upstream of an identified ORF start codon [36,37]. Genomic guanine–cytosine (GC) content was found using the European Molecular Biology Open Software Suite (EMBOSS) geecee program [38,39] and tRNA genes were detected using ARAGORN version 1.2.41 [40,41]. PhageTerm on the Galaxy@Pasteur platform was used to determine the genome termini of SU7 [42,43].

### 2.9. Phylogenetic Tree Construction

Phylogenetic analyses of the SU7 genome were conducted using complete nucleotide sequence alignments. Sequences for whole genomes were obtained from the NCBI genome databases (Table 1). The genome nucleotide sequence of SU7 was aligned against genomes showing E-values = 0 in discontinuous MegaBLAST searches against the nucleotide collection databases restricted to *Caudovirales* phages. Alignments were made in ClustalW with the default setting within the Mega-X software and in MAUVE version 20150226 with default settings [44,45,46]. An unrooted neighbor-joining phylogenetic tree was constructed using Mega-X with default settings. No outgroup was used for tree construction. Node confidence was evaluated using bootstrap testing based on 500 random re-samplings.

## 3. Results and Discussion

### 3.1. SU7 Plaque Morphology, Virion Morphology, Infection Kinetics, and Host Range

**Figure 1:**
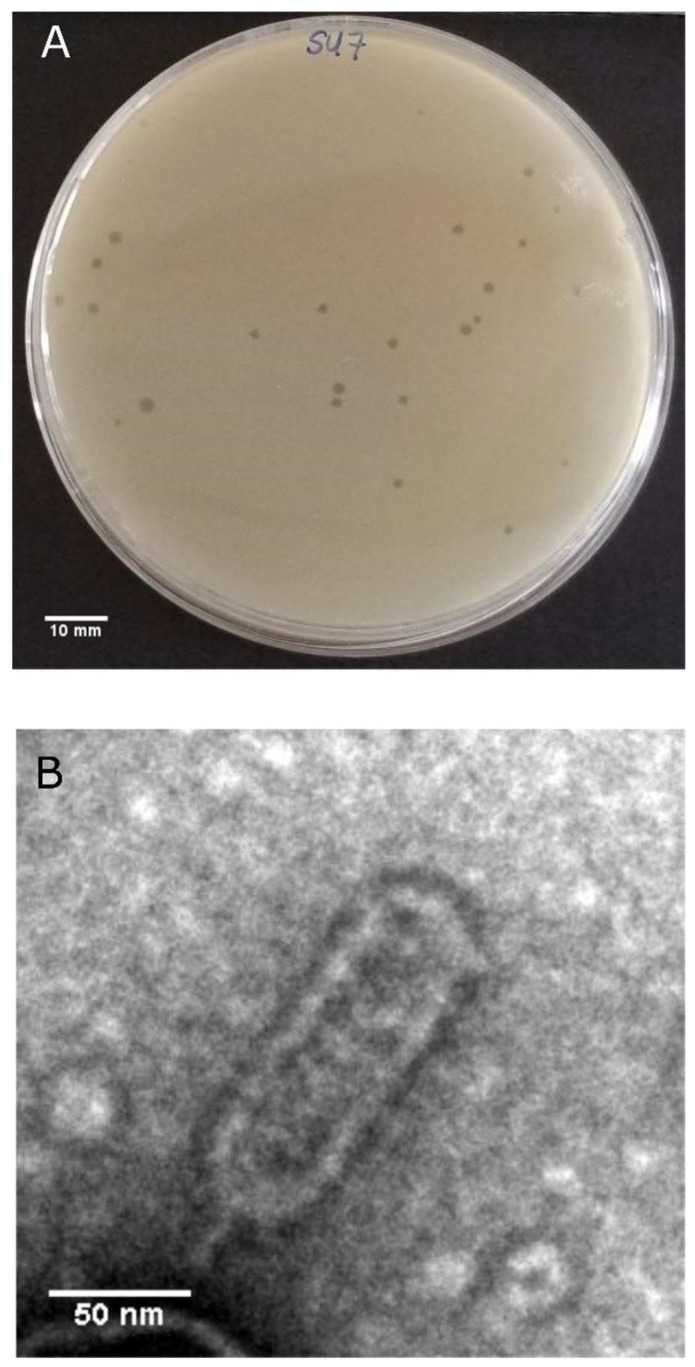
(**A**) Plaque morphology of SU7. Phages were cultured on ETEC7 forming 1 mm clear transparent plaques (1.4 ± 0.3 mm in diameter). (**B**) Transmission electron microscope micrograph of negatively stained phage SU7 at 13,000× magnification. SU7 has an elongated head with a length of 134 nm (134 ± 6 nm) and a width of 44 nm (44 ± 2 nm).

**Figure 2:**
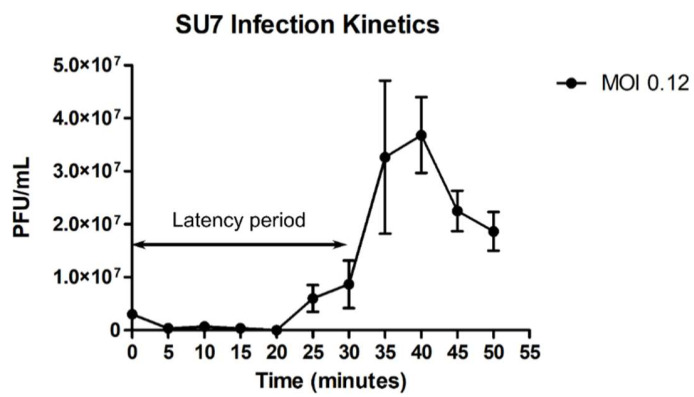
One-step growth curve of phage SU7 infecting bacterial ETEC7 at an MOI of 0.12. After one round of infection, SU7 has a latency period of 30 min, a burst size of 12 PFU/cell, and an adsorption rate constant of 8.78 × 10^−9^ mL/min five minutes post infection. Data points represent the mean of three independent experiments and error bars represent the standard error of each run.

SU7 forms transparent, circular plaques approximately 1 mm in diameter (1.4 ± 0.3 mm) upon a lawn of its host bacteria ETEC7 (Figure 1A). TEM confirms that SU7 is a phage belonging to the rare C3 morphotype of the *Podoviridae* family within the *Caudovirales* order. It has an elongated head, with a length of 134 nm (134 ± 6 nm) and width of 44 nm (44 ± 2 nm), and a very short non-contractile tail, which could not be measured (Figure 1B). Occurring in less than 1% of *Podoviridae* virions, this rare morphotype has been observed among phages belonging to the *Kuravirus* genus (previously referred to as PhiEco32viruses) and include the *E. coli* phages vB_EcoP_SU10, Paul, and phiEco32 [47,48,49,50]. At an MOI of 0.12, SU7 has a latency period of 30 min (defined as the time from the addition of phages to a significant rise in phage concentration) and a burst size of 12 PFU/cell (Figure 2 and Supplementary Figure S1). The adsorption rate constant five minutes post infection was determined to be 8.78 × 10^−9^ mL/min. C3 phages with larger elongated heads theoretically would have smaller plaque morphology (presumably due to the fact that larger virions would diffuse more slowly through the OA compared to phages with smaller heads) as well as lower virulence and inferior infection kinetics [51,52]. Morphology-based and infection kinetic assumptions of SU7 alludes that, like other C3 phages, it would not be an ideal standalone candidate for phage therapy as it is not very virulent, has a long latency period, and poor adsorption [51,53,54].

When tested against 137 *E. coli* strains, phage SU7 showed a narrow host range, infecting 11 out of 137 (8%) test strains (Supplementary Figure S2). Only the host strain ETEC7 displayed confluent lysis. Despite having 90% shared identity (Table 1), C3 phage vB_EcoP_SU10 was capable of lysing 30 out of 72 (42%) of the ECOR collection [51], whereas SU7 was only capable of lysing 9 out 72 (13%). One possible explanation for SU7’s narrow host range could be its host receptor. The most common phage-host receptor for *Podoviridae* coliphages is the O-polysaccharide antigen of the lipopolysaccharide layer [55,56,57], which is highly variable in the *Enterobacter* genus [58]. It should be noted that despite being a rapid, simple, and quantitative screen, single high titer spot testing to determine host range may fail to discriminate between a phage’s ability to replicate within a host and its ability to kill the host strain, as well as result in an overestimation of phage host range and virulence [51,59,60]. From a therapeutic point of view, a narrow host range limits the ability of SU7 to a small set of potential ETEC pathogens [61].

### 3.2. SU7 Genome Characterization

Sequencing revealed SU7 has a double-stranded DNA genome of 76,626 bp in length and a GC content of 42%.

**Figure 3:**
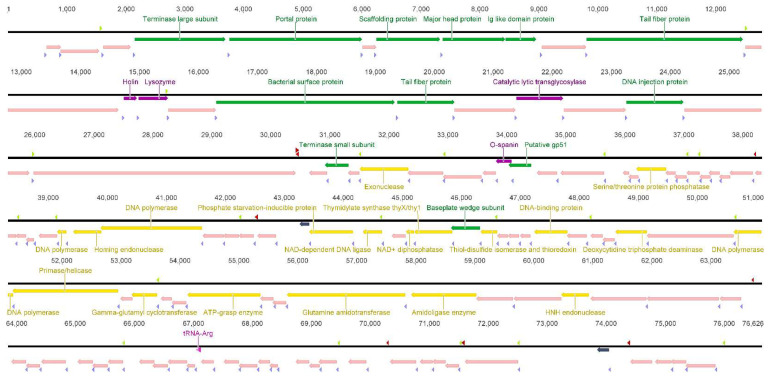
Genome organization with Prokka-predicted ORFs. Direction and annotation colored according to the function group of the genes: yellow, DNA metabolism; light pink, hypothetical proteins; dark gray, proteins with no significant similarity; green, structural proteins; dark purple, lysis related proteins; and pink, tRNA. Transcripts were identified by SoftBerry BROM and PhagePromoter on the Galaxy@GalaxyDockerBuild platform as starting at the promoter regions (light green) and ending at the rho-independent transcription terminators (red), which were identified using ARNold. Putative ribosomal binding sites (light purple) were identified in untranslated regions upstream of the ORFs. Genome visualization was obtained using Geneious software version 6.1.8. See Section 2 for references to the computer programs used.

Genome analyses revealed SU7 has 22 transcripts initiated at hypothetical *E. coli* σ^70^ and phage promoters, eight putative rho-independent transcription terminator sites, 118 ORFs, and a putative tRNA^Arg^ gene. Many phages, including vB_EcoP_SU10, encode for this particular tRNA as way of compensating for low levels of host tRNA and to boost their translation [62]. Putative function was assigned to 30% of the predicted ORFs, which were categorized as either proteins pertaining to DNA metabolism, lysis, or structure (Figure 3 and Supplementary Table S1), whereas 70% matched hypothetical proteins or protein domains with no significant similarity. On average, *Kuravirus* phages with the C3 morphotype tend to have a double-stranded DNA genome with a size of 76,213 bp, which encodes for approximately 122 genes, and have a GC content of 42% (Table 1) [63]. The genome of SU7 did not contain a gene encoding phage RNA polymerase (RNAP), suggesting that the transcription of the phage genome is carried out by the host bacteria machinery only. The presence of phage-specific promoters found by the PhagePromoter program, on the other hand, suggests that the RNAP is modified to accept and utilize these phage promoters instead. In the case a phage RNAP cannot be found, an alternative sigma factor must be encoded by the phage [64]. It is possible that this alternative sigma factor can be found within the genome’s 70%, presenting as a hypothetical protein; however, functional studies are required to make this conclusion. Like other phages presenting the C3 morphotype, there is a lack of correlation between the size of SU7’s genome and head length, as determined by TEM, which suggests that the length of SU7’s DNA does not affect the elongation of it head [49].

PhageTerm predicted the SU7 genome termini ends in short direct terminal repeats (DTR). Like phage PhiEco32, it is possible that SU7 has a replication strategy whereby the formation of either circular or linear concatemeric DNA during infection results in the duplication of its genome ends; thus, the beginning and ending have the same sequence repetition [47]. Upon inspection, SU7’s DTR region is only 53 bp, which is quite short in comparison to other *Kuraviruses* (most being 193 bp). It is possible that the DTR region of SU7 could be longer if more mismatches were allowed during the PhageTerm searches.

### 3.3. Phylogenetic Analyses

Whole genome MegaBLAST alignments of SU7’s nucleotide sequence suggests that SU7 is most similar to *E. coli* phages ECBP2 (94%), EK010 (93%), vB_EcoP_EcoN5 (91%), and vB_EcoP_SU10 (90%) (Table 1 and Supplementary Figure S3).

**Figure 4:**
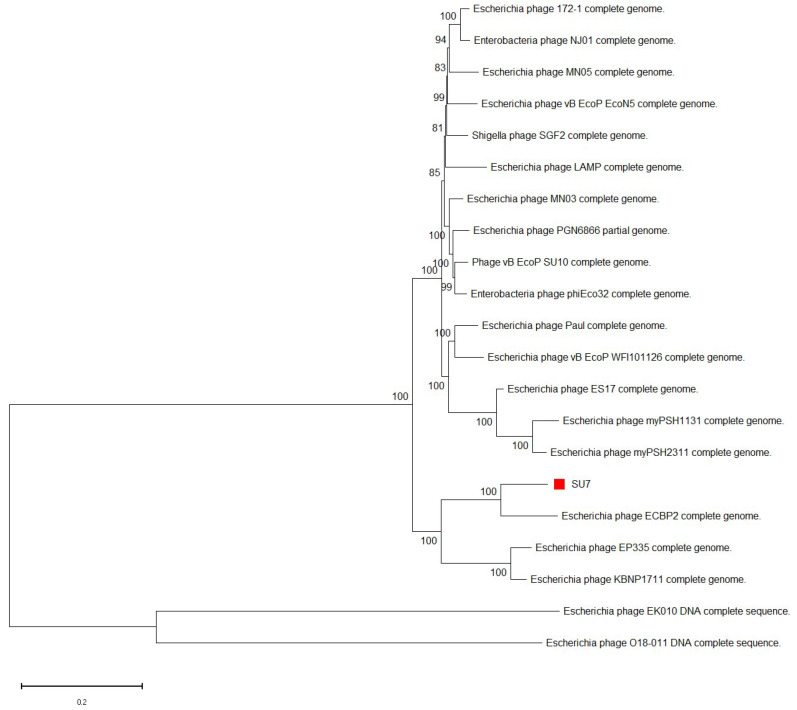
Neighbor-joining phylogenetic tree of phage SU7’s whole genome nucleotide sequence in relation to other *Kuraviruses*. The sequence of SU7 and genome sequences showing an E-value of 0 in discontinuous MegaBlast searches against the nucleotide collection database at NCBI was aligned in ClustalW. The tree was constructed using Mega-X. The nodes depict the bootstrap values, which were calculated based on 500 replicates. The bar represents the number of nucleotide substitutions per site.

All phages that bear genomic similarity to SU7 belong to the *Kuravirus* genus and are morphologically defined by an elongated head (the rare C3 morphotype). Phages belonging to *Kuravirus* share more than 61% DNA sequence and more than 69% protein homology [63]. This can be seen in the clustering pattern in SU7’s phylogenetic analysis based on whole genome nucleotide sequences (Figure 4), suggesting that SU7 is also a *Kuravirus*. References to the *Kuravirus* phages in the phylogenetic analyses and their accession numbers can be found in Table 1.

## 4. Conclusions

Due to their therapeutic potential, phages are ideal candidates to treat antibiotic-resistant infections, such as those arising from ETEC. However, many phages need to be isolated and their genomes characterized before they can be used therapeutically. We have isolated and characterized the genome of vB_EcoP_SU7, a *Podoviridae* phage with the rare C3 morphotype belonging to the *Kuravirus* genus. Successful phage therapy entails the phage(s) given must be virulent and rapidly reproduce, ideally having a short latency period and large burst size, be small in size to allow for better diffusion and site-directed dosage, and have a broad host range [11]. Given the limited host range, their size, and infection kinetics, it is unlikely that C3 phages such as SU7 can be used in a therapeutic way as a standalone treatment and would have to be given in combination with other smaller lytic phages. This research can, however, act as a stepping stone to delve further into C3 phage evolution and *Kuravirus* genomic diversity.

## Figures and Tables

**Table 1:** Genome nucleotide comparison of SU7 and the *Kuraviruses* presenting the C3 morphotype.

Phage	Genome Size (bp)	GC Content	No. of Genes	E-Value	Identity (%)	Query Cover (%)	Accession
Escherichia phage ECBP2	77,315	42.4	121	0	93.88	83	JX415536.1
Escherichia phage EK010	78,078	42.1	117	0	93.09	89	LC553734.1
Escherichia phage vB_EcoP_EcoN5	76,083	42.1	128	0	91.26	54	MN715356.1
Escherichia phage vB_EcoP_SU10	77,327	42.1	125	0	89.77	63	KM044272.1
Escherichia phage PGN6866	78,549	42.3	41	0	88.69	63	MT127620.1
Escherichia phage MN03	77,187	42.2	125	0	88.40	57	MT129653.1
Escherichia phage O18-011	75,646	42.1	121	0	87.88	56	LC553735.1
Escherichia virus phiEco32	77,554	42.3	129	0	87.37	61	EU330206.1
Escherichia phage MN05	76,899	42.2	127	0	87.00	52	MT129655.1
Escherichia phage ES17	75,007	42.1	123	0	86.25	56	MN508615.2
Escherichia phage 172-1	77,266	42.0	130	0	86.20	62	KP308307.1
Escherichia phage NJ01	77,448	42.0	109	0	86.18	61	JX867715.1
Escherichia phage myPSH2311	68,712	42.4	89	0	85.67	45	MG976803.1
Escherichia phage LAMP	68,521	42.2	96	0	85.56	54	MG673519.1
Escherichia phage vB_EcoP_WFI101126	77,307	42.1	136	0	85.45	58	MK373770.1
Escherichia phage Paul	79,429	42.0	134	0	85.18	58	MN045231.1
Escherichia phage EP335	76,622	42.5	126	0	84.69	57	MG748548.1
Escherichia phage KBNP1711	76,184	42.4	126	0	83.28	61	KF981730.1
Shigella phage SGF2	76,964	42.3	119	0	77.85	61	MN148435.1
Escherichia phage myPSH1131	76,163	42.3	97	0	75.90	44	MG983840.1

## Data Availability

The datasets presented in this study can be found in online repositories. The genome has been submitted to NCBI and can be found under the GenBank accession number MZ342906.

## Supplementary Materials

The following are available online at https://www.mdpi.com/article/10.3390/microorganisms9081576/s1, Figure S1: One step growth curve of SU7 over an experimental duration of 65 min, Figure S2: Host range analyses on phage SU7, Figure S3: Multiple genome alignment of SU7 and closely related *Kuraviruses* vB_EcoP_SU10, vB_EcoP_EcoN, ECBP2, and EK010 using MAUVE, Table S1: General features and functions of presumed genes from phage SU7.
