# Supplementary material for: Complete Genome Sequence of vB_EcoP_SU7, a Podoviridae Coliphage with the Rare C3 Morphotype
Source: Microorganisms. 2021 Jul 24;9(8):1576. doi: 10.3390/microorganisms9081576 (PMC8399022; doi:10.3390/microorganisms9081576)
Supplement: Supplementary file 1 [file microorganisms-09-01576-s001.zip › microorganisms-1280630-supplementary.pdf]

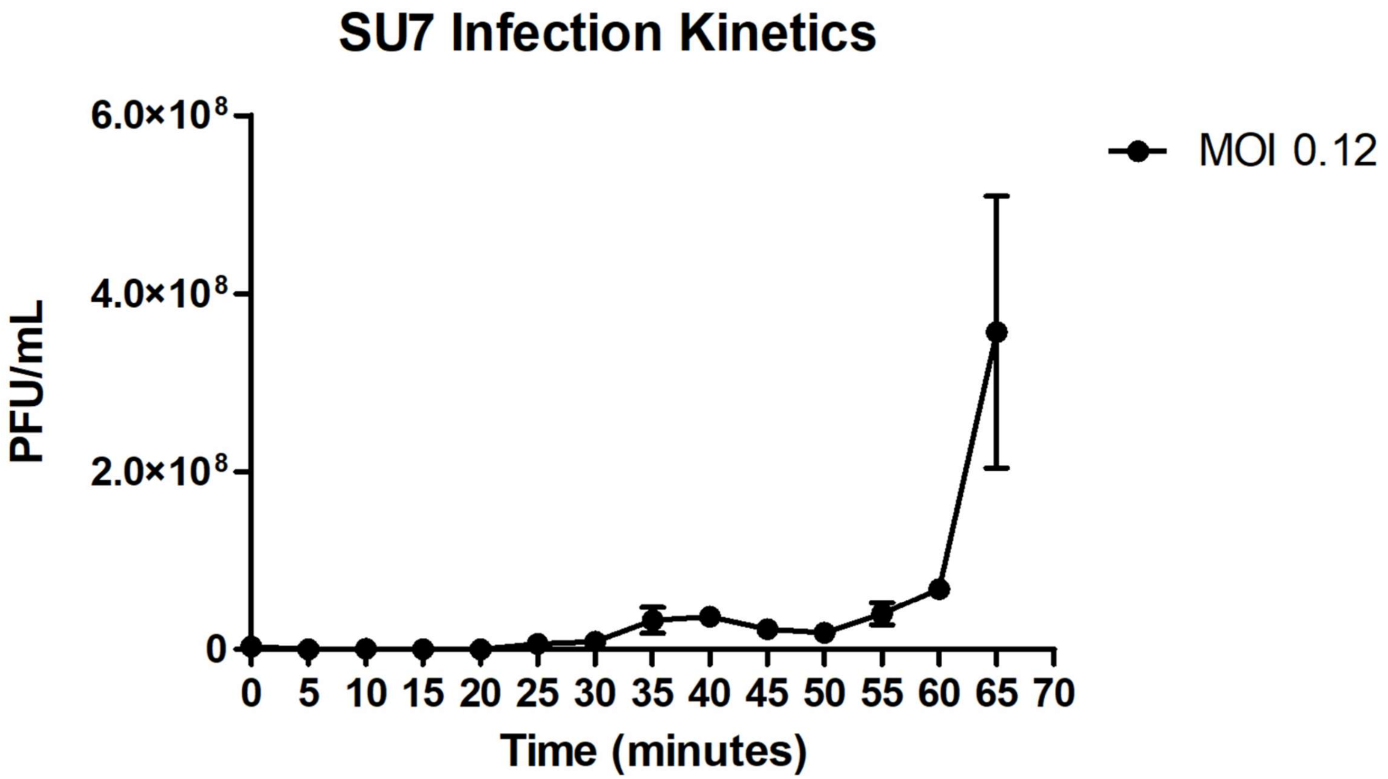

Figure S1. One step growth curve of SU7 over an experimental duration of 65 minutes. Presented is the infection kinetics of SU7 following two rounds of infection. SU7 has a latency period of 30 minutes (depicted as the first rise in phage titer and at 60 minutes) and a burst size of 12 PFU/cell. The adsorption rate constant five minutes post infection is 8.78×10^{-9} mL/min. Data points represent the average of three independent experiments and the error bars represent the standard error.

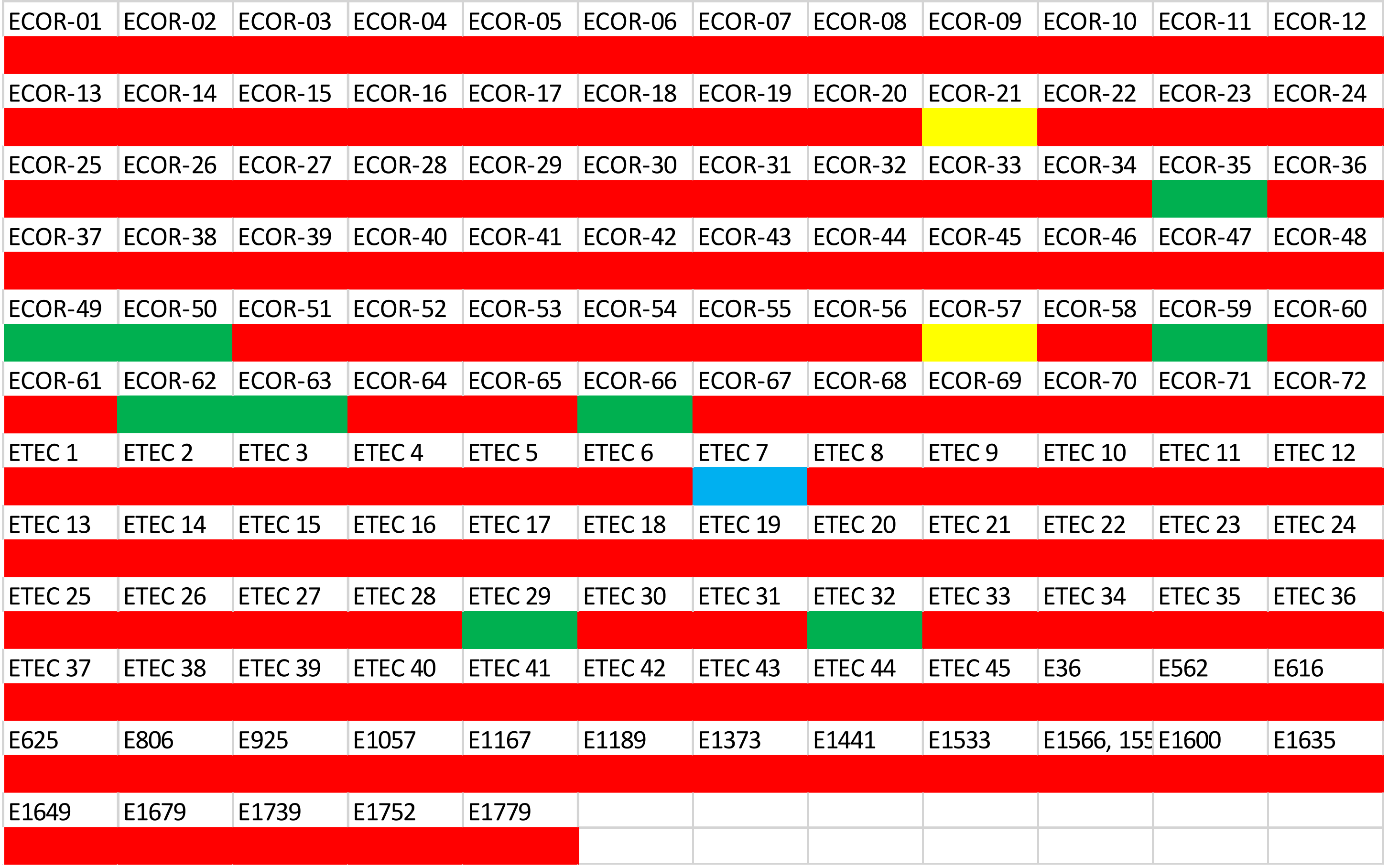

Figure S2. Host range analyses on phage SU7. Data are based on three biological replicates. Bacterial strains used for the host range analyses are ETEC and *E. coli* from the ECOR collection. ■ confluent lysis, ■ individual bacterial colonies in the spot, ■ no effect, and ■ host strain.

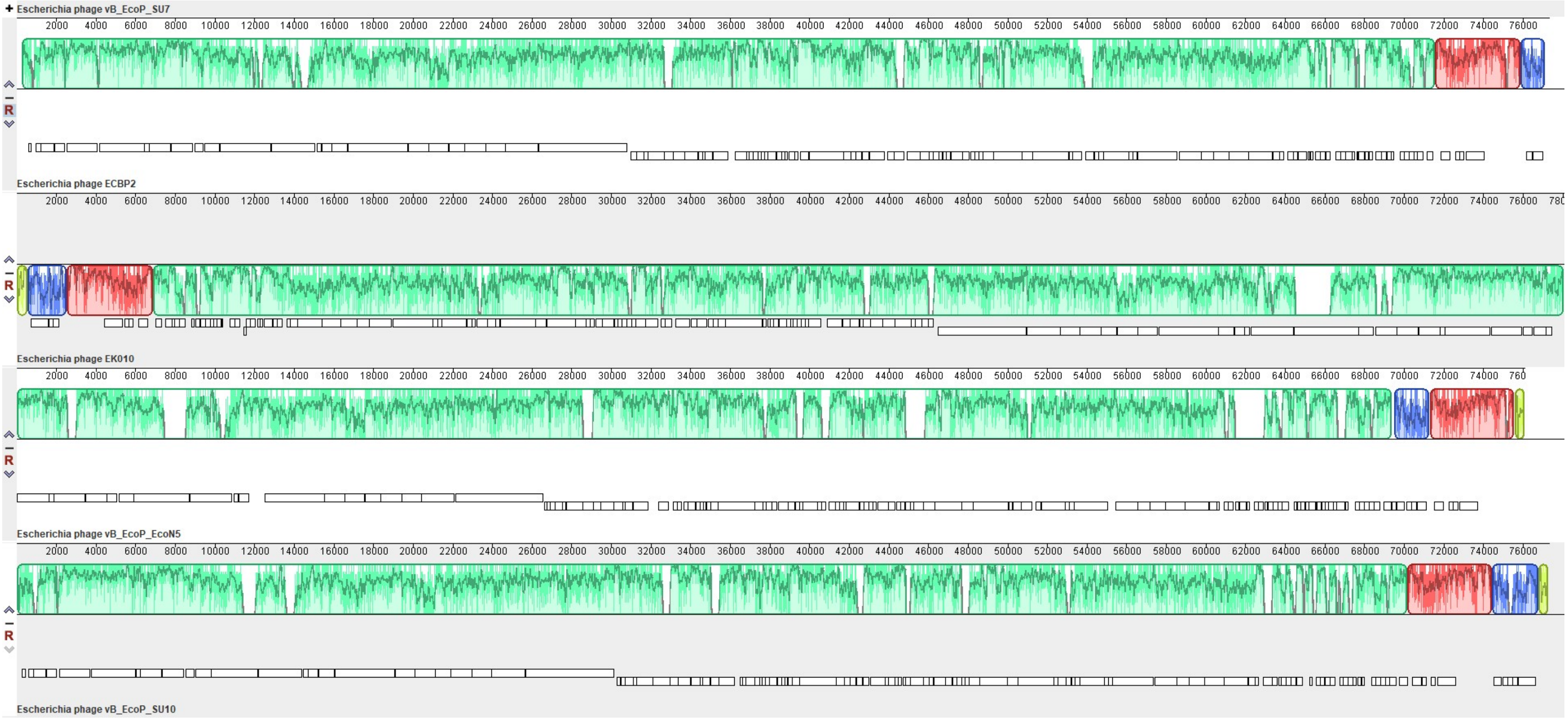

Figure S3. Multiple genome alignment of SU7 and closely related *Kuraviruses* vb_EcoP_SU10, vB_EcoP_EcoN, ECBP2, and EK010 using MAUVE. Bar heights represent the average level of conservation within each region of the genome sequence. White regions represent fragments that have not been aligned or have sequence specific components. Homologous regions within each genome is depicted in different colored blocks.

Table S1. General features and functions of presumed genes from phage SU7. Gene lengths are represented as nucleotides (nt) and the coding strand of DNA where the ORF is located as either top (T) or bottom (B) strand. Identity is the percentage of identical residues in the most similar protein found in the nr NCBI database.

ORFs	Length (nt)	Strand	Predicted function	Query cover	E-value	Identity (%)	Accession
**1**	249	T	Hypothetical protein	98%	5.00E-27	75.00%	BCG45043.1
**2**	654	T	Hypothetical protein	99%	4.00E-138	93.52%	YP_006908854.1
**3**	468	T	Hypothetical protein	89%	6.00E-84	98.57%	BCG45041.1
**4**	1542	T	Terminase large subunit	99%	0	99.42%	BCG45040.1
**5**	2244	T	Portal protein	99%	0	99.06%	BCG45039.1
**6**	237	T	Hypothetical protein	98%	4.00E-40	98.72%	BCG45038.1
**7**	1086	T	Scaffolding protein	99%	0	94.74%	BCG45037.1
**8**	1056	T	Major head protein	99%	0	99.43%	BCG45036.1
**9**	513	T	Ig like domain protein	99%	2.00E-100	99.41%	QGZ13709.1
**10**	753	T	Hypothetical protein	99%	0	98.80%	YP_009152862.1
**11**	2643	T	Tail fiber protein	99%	0	86.73%	QKL16987.1
**12**	2148	T	Hypothetical protein	99%	0	80.19%	QIN95689.1
**13**	219	T	Holin	98%	1.00E-46	97.22%	BCG45031.1
**14**	492	T	Lysozyme	99%	7.00E-119	97.55%	BCG45030.1
**15**	804	T	Hypothetical protein	99%	7.00E-168	91.39%	YP_006908867.1
**16**	3021	T	Bacterial surface protein	99%	0	84.51%	YP_001671764.1
**17**	969	T	Tail fiber protein	99%	0	90.06%	YP_009008712.1
**18**	1032	T	Hypothetical protein	99%	0	90.09%	AVZ45102.1
**19**	798	T	Catalytic lytic transglycosylase	99%	0	94.72%	BCG45025.1
**20**	1053	T	Hypothetical protein	99%	7.00E-162	95.71%	BCG45024.1
**21**	969	T	DNA injection protein	82%	4.00E-132	99.62%	BCG45023.1
**22**	1668	T	Hypothetical protein	99%	0	98.02%	BCG45022.1
**23**	4446	T	Hypothetical protein	99%	0	99.46%	BCG45021.1
**24**	300	B	Hypothetical protein	99%	2.00E-68	95.96%	BCG45019.1
**25**	405	B	Terminase small subunit	98%	1.00E-94	96.99%	BCG45018.1

**26**	192	B	Hypothetical protein	98%	9.00E-41	100.00%	BCG45017.1
**27**	819	B	Exonuclease	99%	0	97.79%	BCG45016.1
**28**	597	B	Hypothetical protein	99%	1.00E-124	85.43%	YP_006908881.1
**29**	651	B	Hypothetical protein	99%	5.00E-160	97.69%	YP_006908882.1
**30**	225	B	Hypothetical protein	98%	4.00E-34	72.97%	YP_006908883.1
**31**	267	B	O-spanin	83%	8.00E-40	82.43%	QEG08131.1
**32**	387	B	Putative gp51	98%	7.00E-58	65.35%	BCG45011.1
**33**	342	B	Hypothetical protein	95%	2.00E-71	90.83%	YP_006908885.1
**34**	741	B	Hypothetical protein	99%	0	98.78%	BCG45009.1
**35**	108	B	Hypothetical protein	97%	5.00E-16	100.00%	QIN96097.1
**36**	165	B	Hypothetical protein	98%	5.00E-32	96.30%	QBQ76467.1
**37**	504	B	Serine/threonine protein phosphatase	99%	6.00E-121	97.60%	YP_009152891.1
**38**	168	B	Hypothetical protein	98%	1.00E-34	100.00%	YP_006908888.1
**39**	204	B	Hypothetical protein	98%	2.00E-17	57.35%	YP_006908889.1
**40**	225	B	Hypothetical protein	98%	4.00E-48	95.95%	YP_006908890.1
**41**	186	B	Hypothetical protein	75%	5.00E-12	100.00%	YP_006908891.1
**42**	180	B	Hypothetical protein	98%	2.00E-36	98.31%	YP_006908892.1
**43**	180	B	Hypothetical protein	98%	3.00E-37	100.00%	BCG45002.1
**44**	393	B	Hypothetical protein	99%	4.00E-93	99.23%	YP_006908894.1
**45**	243	B	Hypothetical protein	76%	2.00E-37	98.39%	BCG45000.1
**46**	168	B	Hypothetical protein	98%	2.00E-34	94.55%	BCG44999.1
**47**	135	B	Hypothetical protein	97%	2.00E-24	95.45%	BCG44998.1
**48**	312	B	Hypothetical protein	99%	8.00E-69	93.20%	YP_006908898.1
**49**	156	B	DNA polymerase	96%	4.00E-31	100.00%	BCG44996.1
**50**	477	B	Homing endonuclease	82%	1.00E-93	98.47%	YP_006908900.1
**51**	1713	B	DNA polymerase	98%	0	98.94%	BCG44996.1
**52**	354	B	Hypothetical protein	99%	7.00E-72	95.73%	BCG44995.1
**53**	258	B	Hypothetical protein	98%	8.00E-60	100.00%	BCG44994.1
**54**	252	B	Hypothetical protein	98%	9.00E-55	98.80%	YP_006908904.1

**55**	318	B	Hypothetical protein	78%	5.00E-50	96.39%	AVP40141.1
**56**	168	B	No significant similarity found				
**57**	741	B	Phosphate starvation-inducible protein	98%	0	99.59%	QPL11101.1
**58**	330	B	NAD-dependent DNA ligase	99%	1.00E-79	100.00%	YP_001671807.1
**59**	234	B	Hypothetical protein	98%	7.00E-51	96.10%	AVP40144.1
**60**	648	B	Thymidylate synthase thyX/thy1	99%	5.00E-146	97.66%	BCG45157.1
**61**	492	B	Baseplate wedge subunit	99%	2.00E-96	90.80%	BCG45156.1
**62**	276	B	Thiol-disulfide isomerase and thioredoxin	98%	4.00E-47	90.11%	BCG44983.1
**63**	177	B	Hypothetical protein	98%	1.00E-36	98.28%	YP_006908913.1
**64**	186	B	Hypothetical protein	98%	2.00E-36	100.00%	YP_006908914.1
**65**	180	B	Hypothetical protein	98%	3.00E-35	98.31%	YP_009008762.1
**66**	576	B	DNA-binding protein	99%	3.00E-140	99.47%	BCG44980.1
**67**	330	B	Hypothetical protein	99%	7.00E-78	100.00%	BCG44979.1
**68**	207	B	Hypothetical protein	98%	1.00E-26	85.29%	BCG44978.1
**69**	198	B	Hypothetical protein	98%	1.00E-41	96.92%	BCG44977.1
**70**	534	B	Deoxycytidine triphosphate deaminase	99%	2.00E-129	99.44%	BCG44976.1
**71**	1473	B	Hypothetical protein	99%	0	93.06%	BCG44975.1
**72**	549	B	DNA polymerase	99%	7.00E-136	99.45%	BCG44974.1
**73**	1791	B	Primase/helicase	99%	0	99.50%	BCG44973.1
**74**	207	B	Hypothetical protein	98%	3.00E-45	100.00%	BCG44972.1
**75**	432	B	Gamma-glutamyl cyclotransferase	98%	3.00E-101	97.18%	BCG44971.1
**76**	186	B	Hypothetical protein	98%	1.00E-37	96.72%	BCG44969.1
**77**	243	B	Hypothetical protein	98%	1.00E-52	98.75%	BCG44968.1
**78**	1236	B	ATP-grasp enzyme	99%	0	96.59%	BCG44967.1
**79**	222	B	Hypothetical protein ECBP2_0080	98%	3.00E-47	94.52%	YP_006908931.1
**80**	234	B	Hypothetical protein ECBP2_0081	98%	4.00E-52	100.00%	YP_006908932.1
**81**	2004	B	Glutamine amidotransferase	99%	0	99.10%	BCG44964.1
**82**	1101	B	Amidoligase enzyme	99%	0	98.63%	BCG44963.1
**83**	627	B	Hypothetical protein	99%	1.00E-152	98.56%	BCG44962.1

**84**	810	B	Hypothetical protein	98%	0	97.75%	BCG44961.1
**85**	471	B	HNH endonuclease	92%	7.00E-43	48.97%	SPP13284.1
**86**	951	B	Hypothetical protein	99%	0	98.42%	BCG44960.1
**87**	1218	B	Hypothetical protein	99%	0	92.84%	BCG44959.1
**88**	363	B	Hypothetical protein	99%	2.00E-82	97.50%	BCG44958.1
**89**	249	B	Hypothetical protein	98%	2.00E-51	91.46%	BCG44956.1
**90**	234	B	Hypothetical protein	98%	8.00E-50	97.40%	BCG44955.1
**91**	414	B	Hypothetical protein	99%	4.00E-97	97.08%	YP_006908943.1
**92**	258	B	Hypothetical protein	52%	8.00E-14	100.00%	BCG44952.1
**93**	264	B	Hypothetical protein	98%	3.00E-60	100.00%	BCG44951.1
**94**	255	B	Hypothetical protein	71%	2.00E-39	100.00%	BCG44950.1
**95**	255	B	Hypothetical protein	98%	2.00E-36	100.00%	BCG44948.1
**96**	255	B	Hypothetical protein	98%	2.00E-56	95.24%	BCG44947.1
**97**	327	B	Hypothetical protein	99%	3.00E-71	94.44%	YP_006908951.1
**98**	141	B	Hypothetical protein	97%	3.00E-23	89.13%	YP_006908952.1
**99**	132	B	NAD+ diphosphatase	97%	3.00E-24	100.00%	YP_009152913.1
**100**	210	B	Hypothetical protein	98%	1.00E-43	97.10%	BCG44946.1
**101**	258	B	Hypothetical protein	98%	4.00E-58	98.82%	BCG44943.1
**102**	312	B	Hypothetical protein	79%	1.00E-54	97.59%	BCG44942.1
**103**	198	B	Hypothetical protein	98%	6.00E-41	98.46%	YP_006908958.1
**104**	141	B	Hypothetical protein	97%	2.00E-27	97.83%	YP_006908959.1
**105**	231	B	Hypothetical protein	98%	1.00E-48	97.37%	BCG44938.1
**106**	177	B	Hypothetical protein	98%	5.00E-36	100.00%	BCG44937.1
**107**	306	B	Hypothetical protein	99%	2.00E-66	98.02%	BCG44936.1
**108**	306	B	Hypothetical protein	99%	6.00E-71	99.01%	BCG44935.1
**109**	444	B	Hypothetical protein	99%	7.00E-105	98.64%	BCG44934.1
**110**	210	B	Hypothetical protein	98%	2.00E-16	55.07%	QEM42684.1
**111**	222	B	Hypothetical protein	98%	1.00E-47	100.00%	BCG44933.1
**112**	225	B	Hypothetical protein	81%	1.00E-37	95.08%	BCG44932.1

**113**	903	B	Hypothetical protein	95%	2.00E-180	95.83%	BCG44931.1
**114**	201	B	No significant similarity found				
**115**	372	B	Hypothetical protein	71%	6.00E-41	66.29%	QIN95674.1
**116**	273	B	Hypothetical protein	98%	7.00E-52	83.33%	BCG44930.1
**117**	249	B	Hypothetical protein	84%	3.00E-33	94.29%	BCG44929.1
**118**	501	B	Hypothetical protein	99%	3.00E-102	88.55%	BCG44928.1
**119**	76	B	$tRNA^{Arg}$				
